# Freshwater transport between the Kara, Laptev, and East-Siberian seas

**DOI:** 10.1038/s41598-020-70096-w

**Published:** 2020-08-03

**Authors:** A. A. Osadchiev, M. N. Pisareva, E. A. Spivak, S. A. Shchuka, I. P. Semiletov

**Affiliations:** 1 Shirshov Institute of Oceanology, Russian Academy of Sciences, Moscow, Russia; 2 Institute of Geology of Ore Deposits, Petrography, Mineralogy and Geochemistry, Russian Academy of Sciences, Moscow, Russia; 3 Moscow Institute of Physics and Technology, Dolgoprudny, Russia; 4 Ilyichov Pacific Oceanological Institute, Far Eastern Branch of the Russian Academy of Sciences, Vladivostok, Russia; 5 National Research Tomsk Polytechnic University, Tomsk, Russia; 6 Northern (Arctic) Federal University, Arkhangelsk, Russia

**Keywords:** Ocean sciences, Physical oceanography

## Abstract

The Kara and Laptev seas receive about one half of total freshwater runoff to the Arctic Ocean from the Ob, Yenisei, and Lena rivers. Discharges of these large rivers form freshened surface water masses over wide areas in these seas. These water masses, i.e., the Ob-Yenisei and Lena river plumes, generate an eastward buoyancy boundary current that accounts for the large-scale zonal freshwater transport along the Siberian part in the Arctic Ocean. In this study we investigate spreading of the Ob-Yenisei plume from the Kara Sea to the Laptev Sea through the Vilkitsky Strait and of the Lena plume from the Laptev Sea to the East-Siberian Sea through the Laptev and Sannikov straits during ice-free season. Large horizontal density gradient between freshened plume water and salty ambient sea water is the main driver of these processes, however, their intensity strongly depends on local wind forcing. The Ob-Yenisei plume is spreading to the Laptev Sea in a narrow alongshore current which is induced by strong and long-term southwesterly winds. Under other wind forcing the plume does not reach the Vilkitsky Strait. The Lena plume is almost constantly spreading to the East-Siberian Sea as a large-scale surface water mass which intensity is governed by eastward Ekman transport and is prone to large synoptic variability.

## Introduction

The Ob, Yenisei and Lena rivers contribute large volumes of freshwater discharge into the Kara (~ 1,500 km^3^ annually from the Ob and Yenisei rivers) and Laptev (~ 800 km^3^ annually from the Lena River) seas that account for approximately one half of the total river runoff into the Arctic Ocean^1–3^. Most of the continental runoff to the Kara and Laptev seas is discharged during ice-free period in June–September and forms sea-wide Ob-Yenisei and Lena river plumes which are among the largest freshwater reservoirs in the Arctic Ocean ^4–6^. River plumes are freshened surface-advected water masses, which seasonally form a relatively thin surface layer, compared to ambient saline sea. As a result, dynamics of river plumes is buoyancy-driven and wind-driven^7–12^, which is also true for large Ob-Yenisei and Lena plumes^3,13–18^. It was previously investigated, that in the absence of strong wind forcing the Coriolis force and large salinity gradient between river plumes and ambient shelf water induce a baroclinic flow along the coast, which was previously addressed in many studies^7,19–21^. This flow can be favored or impeded by local wind forcing. The associated eastward freshwater transport along large segments of northern shores of Eurasia and North America is an important part of large-scale freshwater transport pathways in the Arctic Ocean^22–27^. Eastward alongshore spreading of the Ob-Yenisei and Lena plumes as a buoyancy-driven boundary current was described as a part of the Siberian Coastal Current (SCC)^22^ which is the Eurasian branch of the Riverine Coastal Domain (RCD)^26^.

However, only few previous studies specifically addressed spreading of these river plumes from their source sea to an eastward sea, i.e., transformation of large river plumes to a part of RCD. Based on historical hydrographic data and atmospheric reanalysis, *Dmitrenko *et al*.*^13,28^ revealed dependence of long-term and inter-annual variability of propagation of the Lena plume from the Laptev Sea to the East-Siberian Sea on atmospheric vorticity on quasi-decadal timescales. *Janout *et al*.*^25^ analyzed eastward spreading of the Ob-Yenisei plume from the Kara Sea through the Vilkitsky Strait using numerical modelling and showed its relation to inter-annual variability of atmospheric pressure patterns during summer periods. However, to the extent of our knowledge, synoptic variability of these processes has not been described and discussed before, as well as there is a lack of analysis of direct thermohaline measurements of spreading of the Ob-Yenisei plume through the Vilkitsky Strait.

**Figure 1:**
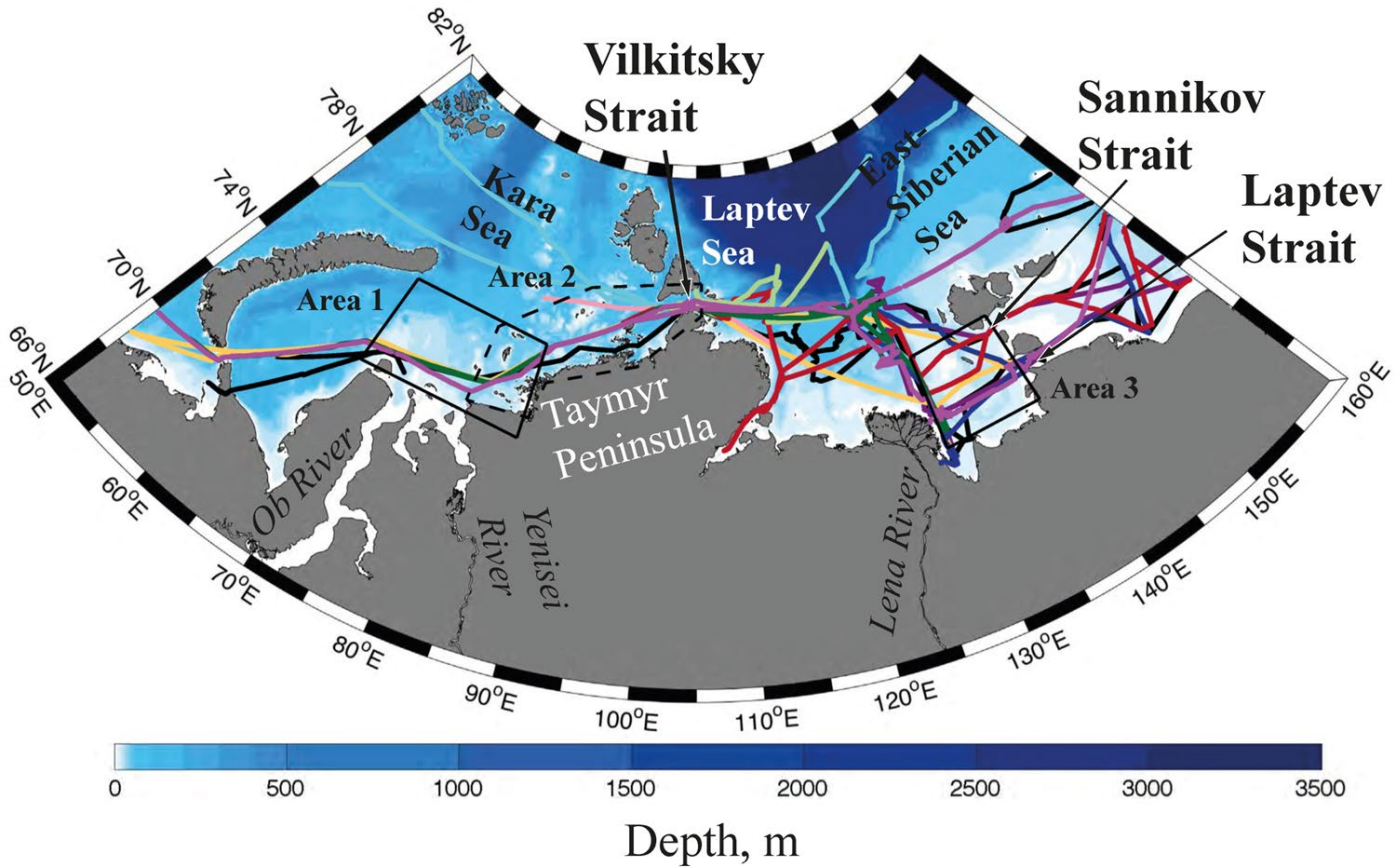
The bathymetry of the Kara, Laptev, and East-Siberian seas and ship tracks of oceanographic surveys (colored lines). Black contours at the Kara and Laptev seas show areas for averaging of the wind forcing (Area 1—the Ob-Yenisei plume, Area 2 (dashed contour)—western coast of the Taymyr Peninsula, Area 3—the Lena plume) used in this study.

In this study we focus on propagation of the Ob-Yenisei plume from the Kara Sea to the Laptev Sea through the narrow Vilkitsky Strait (50–60 km wide) located between the Severnaya Zemlya archipelago and the Taymyr Peninsula (Fig. 1). Also we address propagation of the Lena plume from the Laptev Sea to the East-Siberian Sea through the narrow Laptev (45–55 km wide) and Sannikov (50–60 km wide) straits located in the New Siberian Islands archipelago (Fig. 1). Using in situ hydrographic data, satellite imagery, and atmospheric reanalysis fields, we reveal quick response of these processes to synoptic variability of wind forcing. The obtained results allow assessing synoptic variability of freshwater transport from the Kara Sea to the Laptev Sea during ice-free periods in 1979–2019.

## Results

### Ob-Yenisei and Lena plumes

Continental discharge to Kara and Laptev seas is initially accumulated in the Ob-Yenisei and Lena plumes (however, discharges of many smaller rivers also contribute to these plumes) which extend over hundreds of kilometers in zonal and meridional directions^15,16,29,30^. The subsequent large-scale transport of freshwater discharge is governed by inter-basin spreading of these river plumes which can occur in two possible directions—northwards to the deep Arctic basin and eastwards along the Siberian coast^24,26,27^. The first one is studied mainly by numerical modelling (e.g.,^23,27,30–32^ due to the lack of in situ measurements at the continental slope and deep parts of the Kara and Laptev seas caused by remoteness and very short or absent ice-free period in these areas. Coastal areas in the Kara, Laptev, and East-Siberian seas, which are potentially influenced by eastward spreading of these river plumes, are much better covered by in situ measurements. In this study we emphasize thermohaline measurements in the Vilkitsky, Sannikov, and Laptev straits that play the role of “narrow gates” for eastward inter-basin freshwater transport between the Kara, Laptev, and East-Siberian seas (Fig. 1). We analyze in situ data collected during 15 oceanographic surveys in the Kara, Laptev, and East-Siberian seas in 1999–2019; satellite observations of these seas during cloud-free periods acquired by Terra/Aqua Moderate Resolution Imaging Spectroradiometer (MODIS); wind data from ERA5 atmospheric reanalysis.

### Freshwater transport through the Vilkitsky Strait

**Figure 2:**
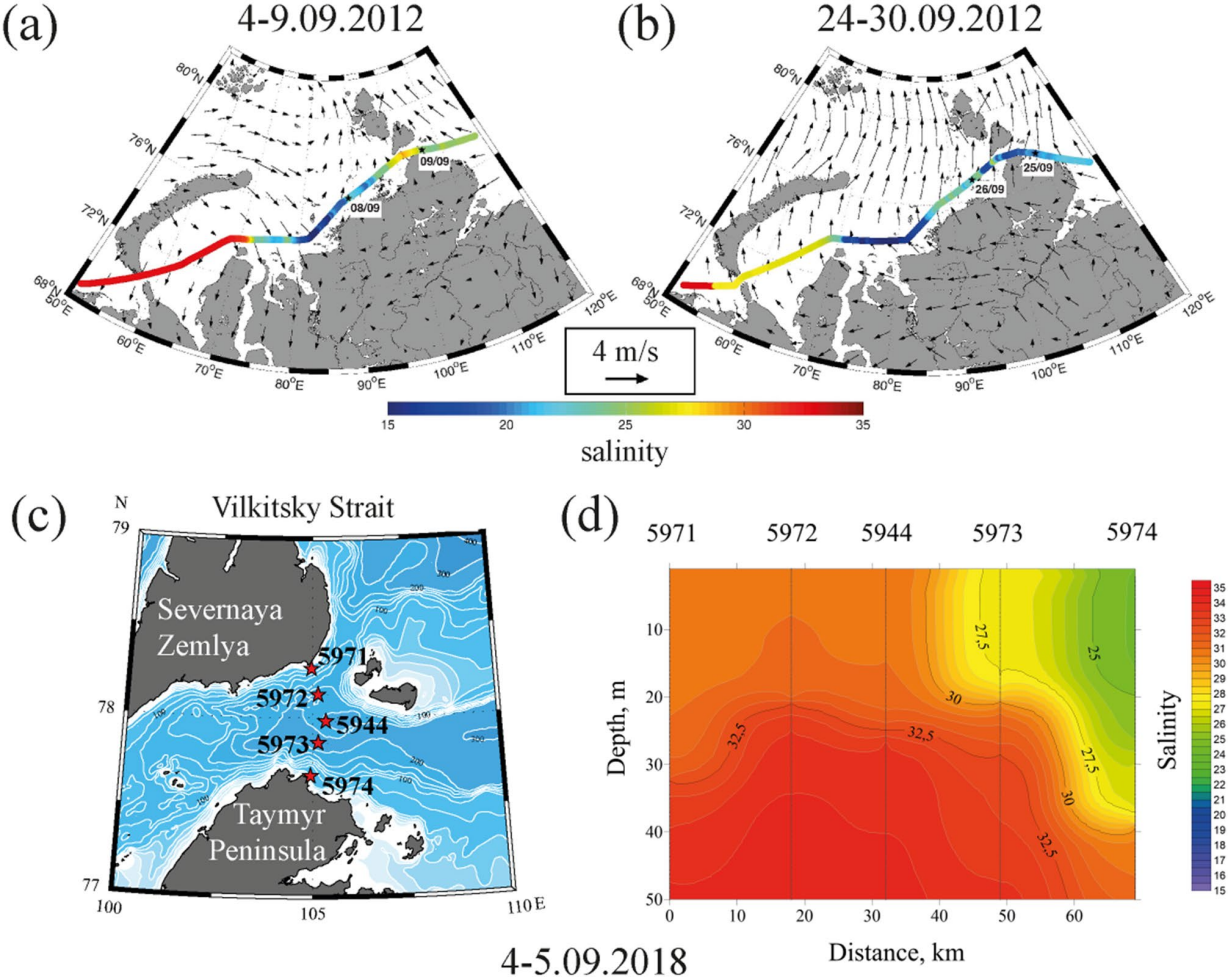
Surface salinity (in color) along the survey tracks in the Kara Sea and 26-days averaged wind (arrows) prior to measurements in the Vilkitsky Strait on 8 September 2012 (**a**) and 25 September 2012 (**b**). Locations of the hydrographic stations (**c**) and the vertical salinity structure across the Vilkitsky Strait (**d**) on 4–5 September 2018.

**Figure 3:**
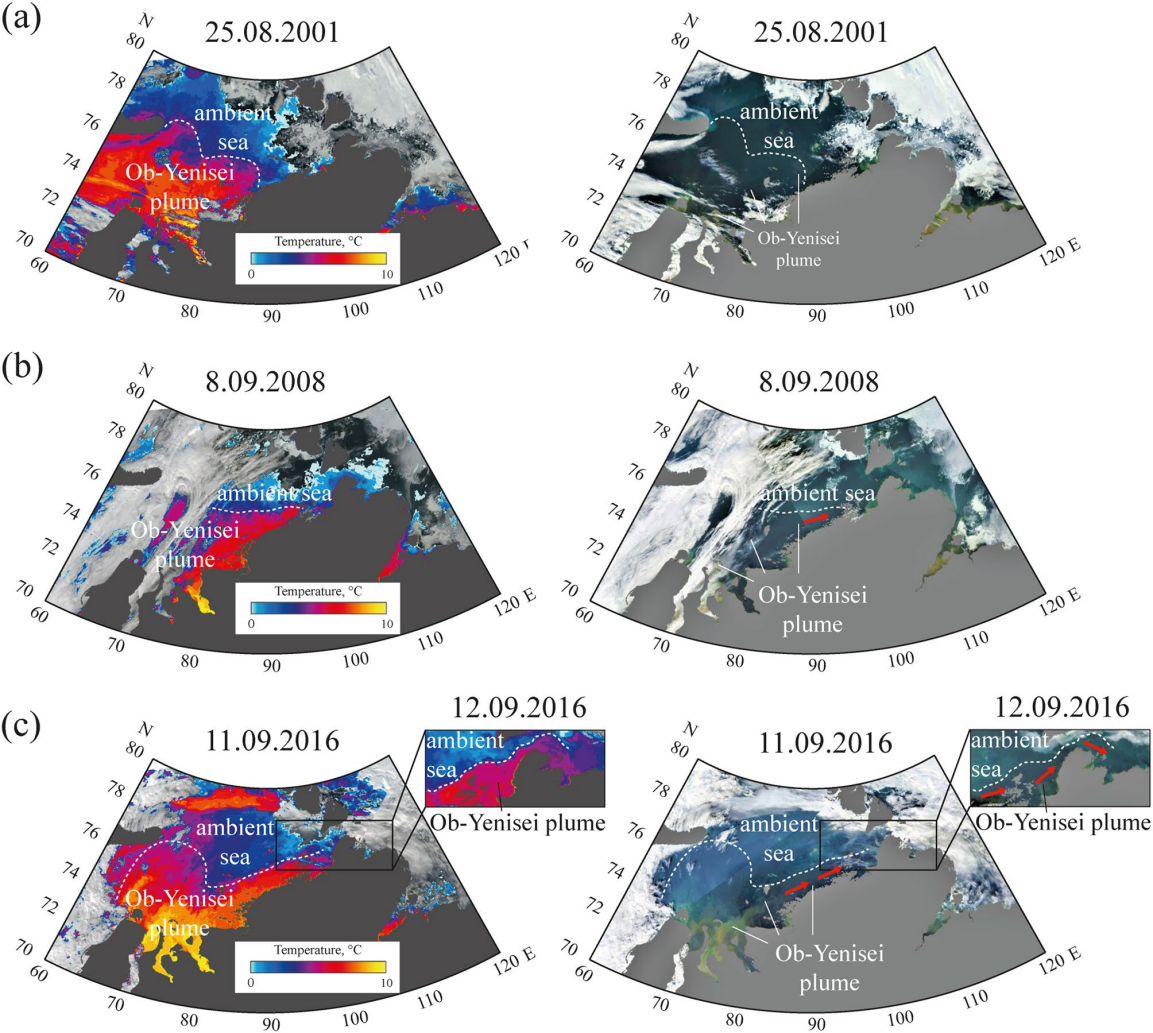
SST (left) and corrected reflectance (right) from MODIS Terra and MODIS Aqua satellite images of the Kara Sea acquired on 25 August 2001 (**a**), 8 September 2008 (**b**), and 11–12 September 2016 (**c**) illustrating different stages of formation of the freshened alongshore buoyancy current (indicated by red arrows) from the core of the Ob-Yenisei plume to the Vilkitsky Strait. Satellite imagery were created using the ESA BEAM software (version 5.0) https://www.brockmann-consult.de/cms/web/beam/releases.

**Figure 4:**
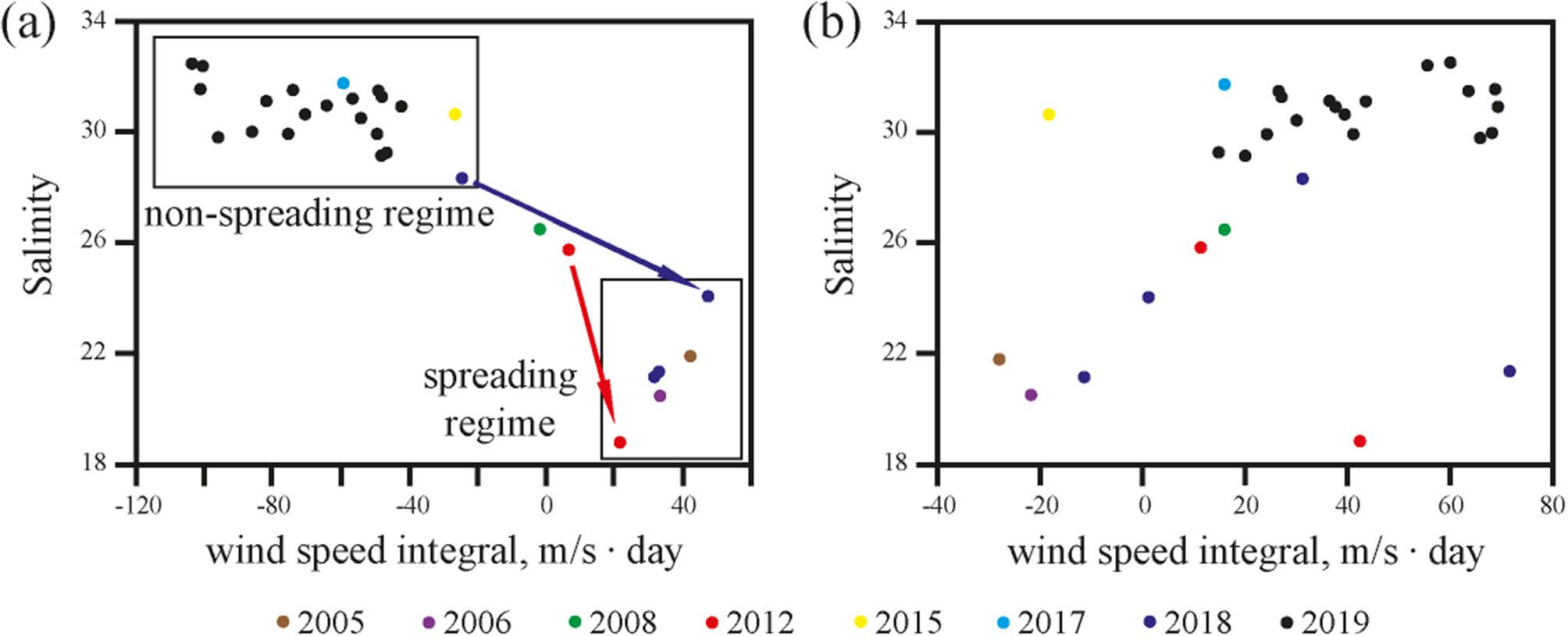
Dependence between alongshore (**a**) and cross-shore (**b**) wind speed integrals and salinity in the Vilkitsky Strait based on in situ measurements from 8 different years (see legend). Wind speed values were integrated over the period of 26 days and averaged over Area 2 shown in Fig. 1. Boxes indicate different regimes in the Vilkitsky Strait, namely, spreading and non-spreading of the alongshore freshwater current through the strait. Arrows in panel (**a**) illustrate response of salinity on synoptic variability of wind forcing observed in September 2012 (red arrow) and in August–September 2018 (blue arrow).

In situ measurements and satellite observations reveal significant inter-annual and synoptic variability of salinity and temperature in the surface layer in the Vilkitsky Strait indicating variability of freshwater transport from the Kara Sea to the Laptev Sea (Figs. 2, 3, 4). In situ data collected in the Vilkitsky Strait during nine oceanographic surveys in 2005–2019 show that surface salinity in the strait varies in a wide range from 19 to 33 (Fig. 4). In 25 cases out of 32 measurements performed during different days in 2005–2019 surface salinity in the strait was > 29 indicating non-spreading of the Ob-Yenisei plume from the Kara Sea to the Laptev Sea. Low salinity, typical for the Ob-Yenisei plume (< 25) was registered in the Vilkitsky Strait only during seven days, namely, on 7 September 2005, 29 September 2006, 25 September 2012, 4–5 September 2018, 26 September 2018, and 19 October 2018.

In situ measurements in the surface layer along the ship track performed in the beginning of September 2012 showed typical salinity structure in the Kara Sea and the Vilkitsky Strait (Fig. 2a). The lowest salinity (< 25) was registered in the central part of the Kara Sea adjacent to the Ob and Yenisei gulfs that indicates location of the core of the Ob-Yenisei plume. Surface salinity in the Vilkitsky Strait was 28–30, therefore, the Ob-Yenisei plume did not propagate through the strait to the Laptev Sea. Figure 2b illustrates the opposite situation, i.e., a spreading event of the Ob-Yenisei plume to the Laptev Sea, observed by in situ measurements at the end of September 2012. Similar to the previous case, the lowest values of surface salinity (< 20) indicate location of the core of the Ob-Yenisei plume in the central part of the Kara Sea. However, surface salinity also was low (< 25) along the coast up to the Vilkitsky Strait, in the strait itself, and further eastward in the western part of the Laptev Sea. Due to absence of large rivers that inflow to the Kara and Laptev seas from the Taymyr Peninsula, the observed reduced surface salinity along the Taymyr Peninsula provide an evidence of spreading of the Ob-Yenisei plume to the Laptev Sea.

The vertical structure in the Vilkitsky Strait during a spreading event of the Ob-Yenisei plume to the Laptev Sea was measured on 4–5 September 2018 at five hydrographic stations located across the strait (Fig. 2c). The Ob-Yenisei plume propagated as a narrow (~ 20 km) and deep (20 m) buoyancy current along the southern shore of the strait (Fig. 2d). Vertical salinity profile measured at the southern stations showed sharp gradient between well-mixed freshened (24–25) and warm (3–4 °C) Ob-Yenisei plume, on the one hand, and saline (> 30) and cold (− 1 to 1 °C) subjacent sea water, on the other hand (Fig. 2d).

In order to study spreading of the Ob-Yenisei plume to the Laptev Sea we analyzed sea surface temperature (SST) products acquired from Terra/Aqua MODIS satellite data. Large river plumes formed in the Kara and Laptev seas during summer and early autumn are significantly warmer than surrounding sea due to the large temperature difference between river and sea water^33–35^. Due to very low solar radiation in the study areas, surface temperature contrast formed between shallow and deep water is much lower, than the contrast between the plumes and ambient sea^33^. As a result, sharp temperature gradients are formed between the warm and freshened Ob-Yenisei plume, on the one hand, and ambient cold and saline sea, on the other hand, which can be used to distinguish these water masses (Fig. 3). MODIS SST products are effective for tracking the Ob-Yenisei and Lena plumes, however, misleading SST values are observed over clouds, which is a well-known feature of the satellite SST products. For this purpose we provide corrected reflectance MODIS images to show the location of clouds and cloud-free sea areas during the satellite observation periods.

In order to study spreading of the freshened alongshore buoyancy current from the core of the Ob-Yenisei plume in the central part of the Kara Sea towards the Vilkitsky Strait, we analyzed all cloud-free and ice-free satellite images of the study area acquired in 2000–2019. Due to common cloudy weather conditions, we detected only 51 periods (1–4 days long) when the Vilkitsky Strait and the adjacent sea were clearly seen in optical satellite images and SST structure could be identified. In particular, there was no individual event of spreading of this current, which was clearly visible at a sequence of cloud-free images from its initial formation till its propagation through the Vilkitsky Strait. However, the analysis of multiple 1–4 days long sequences of cloud-free images revealed that this process has stable pattern. Different stages of formation and propagation of this current are shown in Fig. 3.

Satellite images of the Kara Sea acquired on 25 August 2001 show that the Ob-Yenisei plume occupied the area in the central part of the Kara Sea and was not spreading eastward from the longitude of 90° E (Fig. 3a). On 8 September 2008 the Ob-Yenisei plume also was located in the central part of the Kara Sea; however, the eastern part of the plume was stretched along the Taymyr Peninsula till the longitude of 100° E (Fig. 3b). This feature shows formation of the warm and freshened eastward alongshore current from the Ob-Yenisei plume, albeit this current did not reach the Vilkitsky Strait. The well-developed alongshore current was observed on 11–12 September 2016, it propagated from the central part of the Kara Sea to the Vilkitsky Strait and further eastward to the Laptev Sea (Fig. 3c). This current was ~ 100 km wide in the eastern part of the Kara Sea, then in the Vilkitsky Strait its width decreased to 15–30 km due to topographic barriers at this area. Sharp temperature gradient between the warm Ob-Yenisei plume and cold ambient sea was evident in the cloud-free satellite image of the Vilkitsky Strait from 12 September 2016 (Fig. 3c).

In order to study the background of formation of freshened alongshore currents from the Kara Sea to the Laptev Sea, we performed joint analysis of ERA5 wind reanalysis data and in situ data collected in the Vilkitsky Strait and the adjacent areas of the Kara and Laptev seas during nine oceanographic field surveys. We analyzed salinity data collected near the southern coast of the Vilkitsky Strait which are indicative of presence or absence of the narrow freshened alongshore current in the strait and do not depend on cross-shore shift and meandering of this current. This analysis revealed direct dependence between atmospheric forcing and freshwater transport in the eastern part of the Kara Sea. All observed periods of eastward spreading of the Ob-Yenisei plume and its propagation through the Vilkitsky Strait (indicated by low salinity) were preceded by periods of strong southwesterly winds over the central and eastern parts of the Kara Sea. On the opposite, different types of atmospheric circulation were observed before non-spreading periods of the Ob-Yenisei plume.

In order to evaluate the influence of wind forcing on eastward spreading of the freshened surface layer we calculated the time integrals of alongshore wind $W_{N}^{x} = \mathop \smallint \limits_{T - N}^{T} u \cdot dt$ and cross-shore wind $W_{N}^{y} = \mathop \smallint \limits_{T - N}^{T} v \cdot dt$, where *u* and *v* are the alongshore and cross-shore components of wind speed, the integration is performed over *N* days preceding the day *T* of in situ measurements. Note that *W*_*N*_ takes into account both the magnitude and the duration of the wind forcing. Then we tested dependence of salinity in the Vilkitsky Strait on value of $W_{N}^{x}$ and $W_{N}^{y}$ averaged over two different areas in the Kara Sea. The first testing area (Area 1 in Fig. 1) covers the central part of the Kara Sea typically occupied by the Ob-Yenisei plume during ice-free periods according to in situ salinity data obtained from the World Ocean Database (WOD)^36^. The second testing area (Area 2 in Fig. 1) was selected along the western coast of the Taymyr Peninsula, i.e., the coastal area between the core of the Ob-Yenisei plume and the Vilkitsky Strait. Also we varied the duration of integration period *N* from 1 to 60 days in order to detect the period of response of spreading pattern of the Ob-Yenisei plume on variability of wind forcing.

Salinity in the Vilkitsky Strait showed significant correlation (*R* > 0.8) with alongshore (projected at an angle 30° counterclockwise to the latitude line) wind integral $W_{N}^{x}$ averaged over Area 2 for integration periods *N* between 18 and 36 days. The maximum value of Pearson correlation coefficient *R* (0.9) corresponds to the integration period of 26 days (Fig. 4a). The correlation between salinity in the strait and cross-shore wind integral $W_{N}^{y}$ averaged over Area 2 was also rather high (*R* = 0.4) (Fig. 4b).

Both alongshore (zonal) and cross-shore (meridional) wind integrals averaged over Area 1 also did not show any relation with salinity in the strait. No relation between wind forcing over Area 1 and salinity in the strait is caused by the following reason. Due to large distance (500 km) between the core of the Ob-Yenisei plume (Area 1) and the Vilkitsky Strait, the plume does not propagate towards the straight as large-scale surface water mass. Instead, it is advected in a narrow quasi-geostrophic alongshore current that induces eastward freshwater transport from the core of the plume towards the strait^21,37,38^ that is supported by in situ data (Fig. 2) and satellite observations (Fig. 3). This current is enhanced by downwelling-favorable winds and halted by upwelling-favorable winds^39,40^, as was particularly described for the large-scale alongshore freshwater transport in the Arctic Ocean^22,26,41^. As a result, wind forcing over the core of the Ob-Yenisei plume (Area 1) does not directly determine freshwater transport between the Kara and Laptev seas, however, it is governed by alongshore wind forcing along the western coast of the Taymyr Peninsula (Area 2). Therefore, salinity in the Vilkitsky Strait shows significant dependence on alongshore wind forcing over the Area 2.

The observed high dependence between salinity in Vilkitsky Strait and cross-shore wind integral over the Area 2 is presumably caused by the following reason. Onshore winds arrest a river plume at the coastal area, while offshore winds tend to detach a plume from the sea coast^9,10^. Therefore, offshore winds induce increased mixing of the Ob-Yenisei plume with ambient sea that results in high salinity in the Vilkitsky Strait. On the opposite, once the Ob-Yenisei plume reached the Vilkitsky Strait, onshore winds favor the plume to remain in the strait. However, this mechanism is secondary in the context of spreading of the Ob-Yenisei plume to the Laptev Sea, as compared to the influence of alongshore winds. In particular, low salinity in the Vilkitsky Strait was observed under any type of cross-shore wind forcing.

In situ measurements performed in September 2012 provide an evidence of response of freshwater transport through the Vilkitsky Strait on synoptic variability of wind forcing along the western shore of the Taymyr Peninsula (Fig. 2a, b) illustrated by red arrow in Fig. 4a. On 8 September 2012 the eastern part of the Ob-Yenisei plume was registered on a distance of 150 km to the west from the Vilkitsky Strait, while salinity in the strait was equal to 26 (Fig. 2a). The period of measurements was preceded by variable wind forcing in the end of August. Moderate southeasterly winds registered during 27–30 August were followed by strong southwesterly winds on 31 August–5 September and southeasterly winds on 6–8 September. As a result, alongshore wind speed integral $W_{26}^{x}$ during this period was moderate (6 m/s day) and the freshened alongshore current spread to the eastern part of the Kara Sea, but did not reach the Vilkitsky Strait. On 9–25 September the local atmospheric circulation was dominated by southerly and southwesterly winds, the strongest southwesterly winds occurred during 22–25 September. The resulting large alongshore wind speed integral (20 m/s day) caused flow of low saline surface layer (18–19) through the Vilkitsky Strait to the Laptev Sea that was registered by in situ measurements on 25 September 2012 (Fig. 2b).

Similar formation of the freshened alongshore current and its propagation through the Vilkitsky Strait in response to strong southwesterly winds were observed at the end of August—beginning of September 2018 (blue arrow in Fig. 4a). Moderate and strong (5–15 m/s) northeastern, eastern, and southeasterly winds dominated the atmospheric circulation in the Kara Sea in the first half of August 2018. As a result, the Ob-Yenisei plume was arrested in the central part of the Kara Sea and surface salinity in the Vilkitsky Strait on 23 August 2018 was 28–29. Moderate and strong (7–14 m/s) southwesterly winds that prevailed in the eastern part of the Kara Sea in the end of August and beginning of September 2018 induced formation of the alongshore freshened (24–25) current which was registered in the Vilkitsky Strait on 5 September 2018.

### Freshwater transport through the Laptev and Sannikov straits

**Figure 5:**
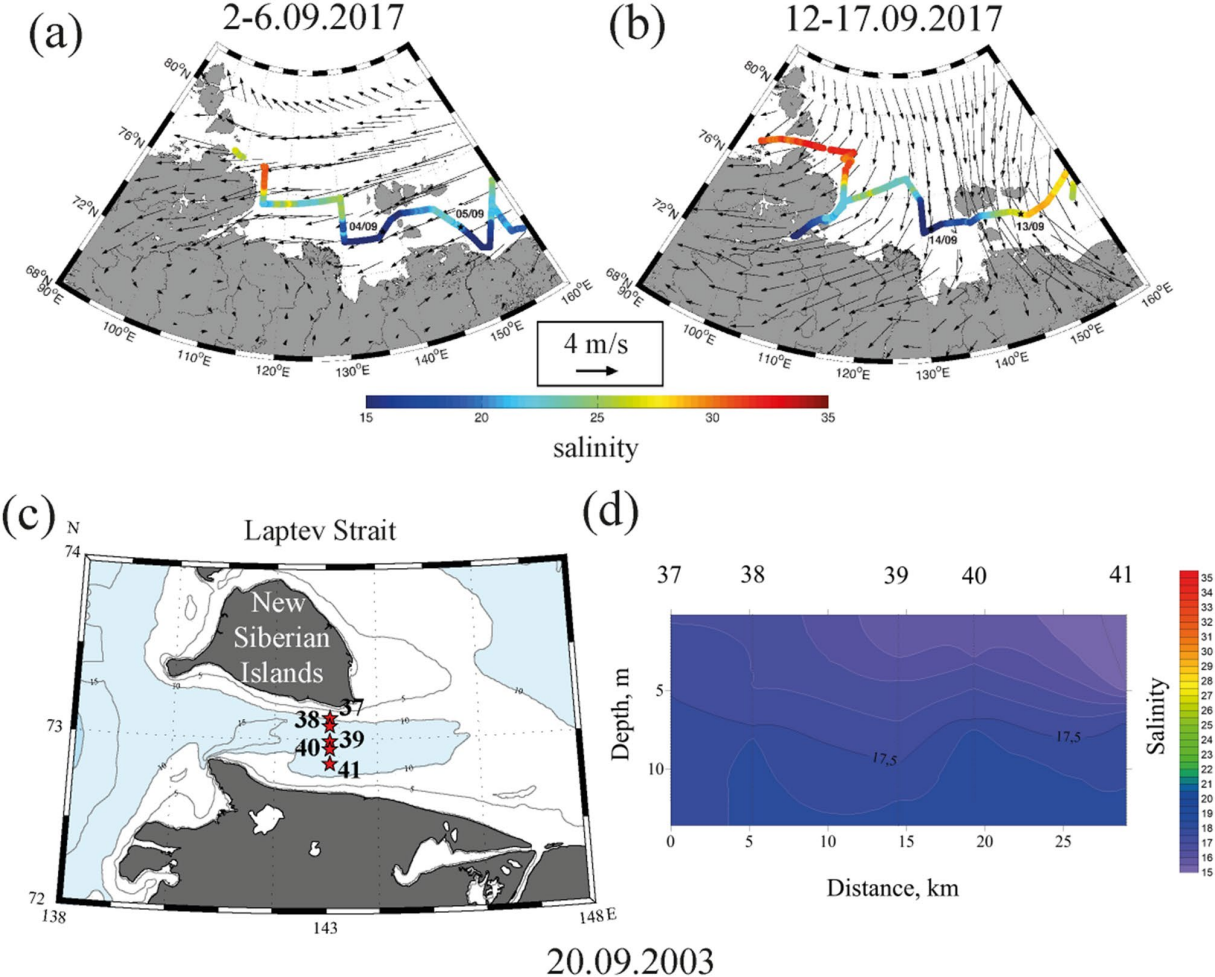
Surface salinity (in color) along the survey tracks in the Laptev Sea and 9-days averaged wind (arrows) prior to measurements in the Sannikov Strait on 4 September 2017 (**a**) and 13 September 2017 (**b**). Locations of the hydrographic stations (**c**) and the vertical salinity structure across the Laptev Strait (**d**) on 20 September 2003.

**Figure 6:**
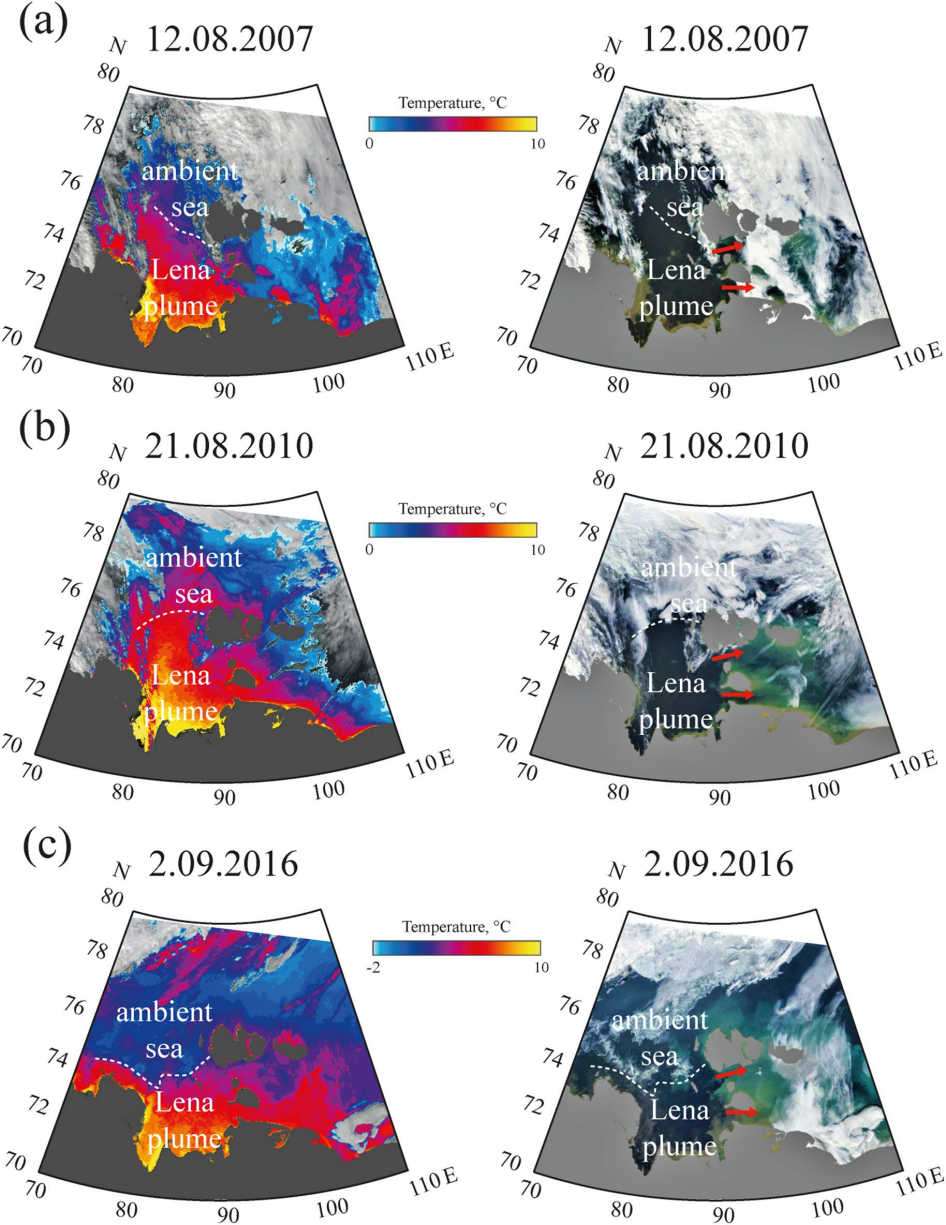
SST (left) and corrected reflectance (right) from MODIS Terra and MODIS Aqua satellite images of the Laptev Sea acquired on 12 August 2007 (**a**), 21 August 2010 (**b**), and 2 September 2016 (**c**) illustrating spreading of the Lena plume to the Laptev and Sannikov straits (indicated by red arrows). Satellite imagery were created using the ESA BEAM software (version 5.0) https://www.brockmann-consult.de/cms/web/beam/releases.

**Figure 7:**
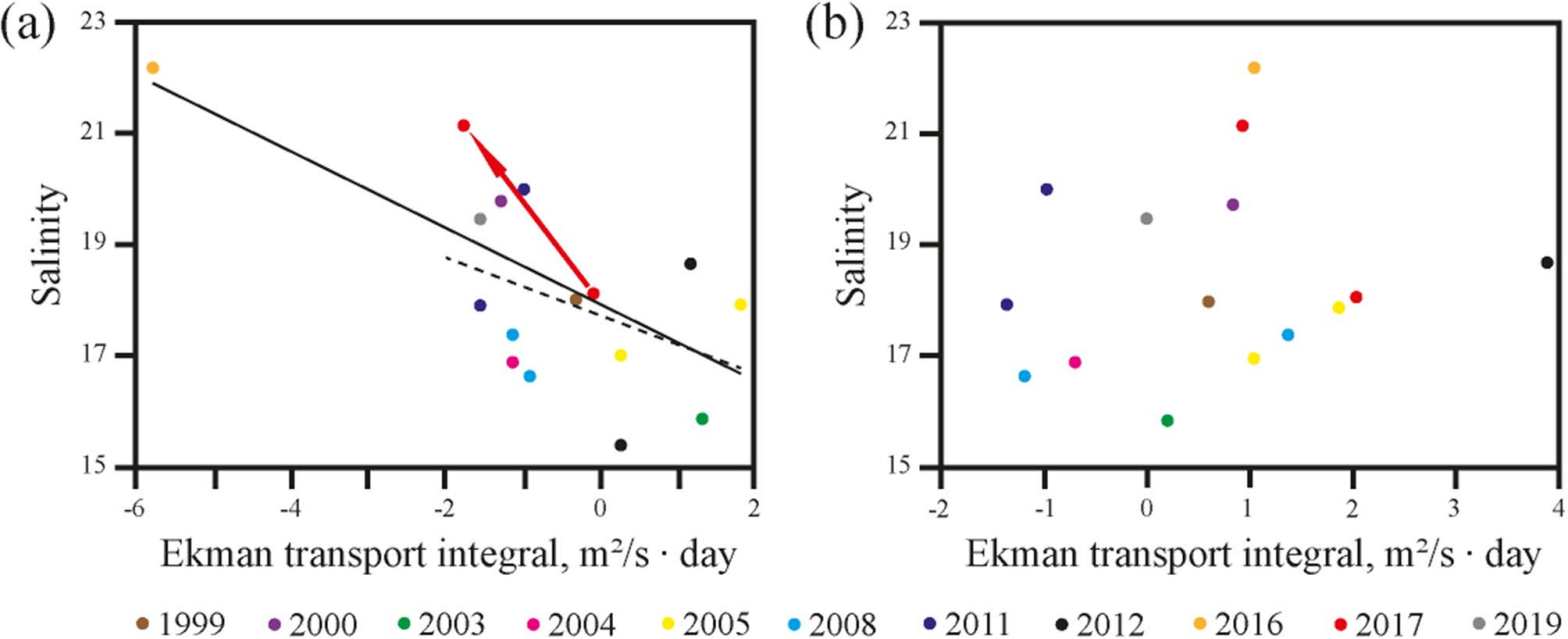
Dependence between averaged zonal (**a**) and meridional (**b**) Ekman transport integral and salinity in the Laptev and Sannikov straits based on in situ measurements from 11 different years (see legend). Ekman transport values were integrated over the period of 9 days and averaged over Area 3 shown in Fig. 1. The black lines in panel (**a**) represent the linear trends calculated for the whole set of points (solid line) and for the set without the outlier (dashed line). Red arrow in panel (**a**) illustrates response of salinity on synoptic variability of wind forcing observed in September 2017.

Freshwater transport from the Laptev Sea to the East-Siberian Sea through the Laptev and Sannikov straits was studied using in situ measurements and satellite observations (Figs. 5, 6, 7). In situ data collected in the Laptev and Sannikov straits during eleven oceanographic surveys in 1999–2019 show that salinity in these straits varied between 16 and 22 (Fig. 7), which is much smaller than in the Vilkitsky Strait (Fig. 4). As a result, the Lena plume was present in the Laptev and Sannikov straits during all periods of field surveys. Synoptic variability of surface salinity in these straits was 1–3, which is associated with variability of position and internal structure of the large Lena plume.

Surface salinity measurements along the ship track showed different salinity structure in the Sannikov Strait and the adjacent area of the East-Siberian Sea in the beginning and in the middle of September 2017 (Fig. 5a, b). During both periods the lowest salinity (< 20) was registered in the south-eastern part of the Laptev Sea between the Lena Delta and the New Siberian Islands indicating location of the core of the Lena plume. In the beginning of September 2017 the Lena plume propagated through the Sannikov Strait and occupied wide area in the adjacent western part of the East-Siberian Sea. In particular, surface salinity was 18 in the Sannikov Strait and did not exceed 25 in the East-Siberian Sea till the longitude of 160° E (Fig. 5a). In the middle of September 2017 surface salinity in this area dramatically changed. The isohaline of 25 shifted to the longitude of 145° E, while salinity in the Sannikov Strait increased to 21–22 (Fig. 5b). The observed increase of surface salinity in the strait and in the western part of the East-Siberian Sea provide an evidence of westward shear of the Lena plume and the related decrease of freshwater transport from the Laptev Sea to the East-Siberian Sea.

Vertical salinity measurements performed at five hydrographic stations on 20 September 2003 (Fig. 5c) showed that the Lena plume occupied the whole water column of the shallow (10–15 m deep) and narrow (50 km wide) Laptev Strait. Surface salinity across the strait varied from 15 to 17 on 20 September 2003, while salinity in the bottom layer did not exceed 18 (Fig. 5d). Vertical salinity measurements performed on 30 August 2008 in the Laptev Strait also showed homogenous salinity structure across the strait. The freshened surface layer with salinity equal to 12–13 was 8–10 m deep, the maximal salinity in the bottom layer was 21.

The eastern part of the Lena Delta, which accounts for 80–90% of freshwater discharge from the Lena River^42^, is located 350–400 km far from the Laptev and Sannikov straits (Fig. 1). As a result, the Lena plume typically occupies large area in the southeastern part of the Laptev Sea adjacent to these straits and is spreading through them to the western part of the East-Siberian Sea that was registered by in situ salinity measurements. The area occupied by the warm Lena plume and bounded by sharp temperature gradient with cold ambient sea can be detected at SST satellite imagery (Fig. 6). Analysis of satellite imagery of the Laptev Sea and the western part of the East-Siberian Sea acquired during cloud-free and ice-free periods in 2000–2019 confirms that the Lena plume is constantly present in the Laptev and Sannikov straits and the eastern boundary of the Lena plume is located in the East-Siberian Sea (Fig. 6). In contrast to the freshwater transport through the Vilkitsky Strait that occurs within a narrow coastal current, freshened surface layer occupies the whole areas of the Laptev and Sannikov straits, i.e., no surface manifestations of cold ambient sea water were registered within these straits. Note that elevated surface turbidity in many coastal areas in the Laptev and East-Siberian seas (including the Laptev Strait) is formed as a result of extremely intense coastal erosion due to active thermal abrasion^43,44^. Turbid regions associated with coastal erosion are adjacent to long segments of sea coast and do not correspond to spreading areas of river plumes^35^. In particular, narrow alongshore stripe of turbid water in the Laptev Strait formed by coastal erosion does not correspond to low-saline waters of the Lena plume, which occupies the whole strait from the southern to the northern coast that is visible at SST images. Corrected reflectance MODIS images are provided in Fig. 6 to show the location of clouds and cloud-free sea areas during the satellite observation periods.

In order to study dependence of salinity in the Laptev and Sannikov straits on wind forcing we calculated the time integrals of wind components $W_{N}^{x}$ and $W_{N}^{y}$ averaged over the area typically occupied by the Lena plume in the southeastern part of the Laptev Sea according to WOD data (Area 3 in Fig. 1). Surface salinity in the straits showed significant linear dependence (*R* > 0.6) on cross-shore (meridional) wind integral $W_{N}^{y}$ calculated for integration periods *N* between 4 and 13 days. On the other hand, no relation was observed between salinity in the straits and alongshore (zonal) wind integral $W_{N}^{x}$. This reveals that meridional wind, i.e. zonal Ekman transport, plays the predominant role in spreading of the Lena plume from the Laptev Sea to the East-Siberian Sea. Due to this fact, we calculated integrals of the zonal Ekman transport $E_{N}^{x} = \mathop \smallint \limits_{T - N}^{T} \frac{{\rho_{a} C_{D} v\sqrt {u^{2} + v^{2} } }}{{\rho_{s} f}}dt$ and the meridional Ekman transport $E_{N}^{y} = - \mathop \smallint \limits_{T - N}^{T} \frac{{\rho_{a} C_{D} u\sqrt {u^{2} + v^{2} } }}{{\rho_{s} f}}dt$, where *ρ*_*a*_ is the density of the air, *ρ*_*s*_ is the density of the sea water, *C*_*D*_ is the drag coefficient prescribed equal to 0.0013, *f* is the Coriolis parameter^45^. These Ekman transport integrals are measures of effectiveness of wind events on currents in the sea surface layer^46^. The largest Pearson correlation coefficient *R* (0.7) between the integral of the zonal Ekman transport $E_{N}^{x}$ averaged over Area 3 and the surface salinity in the Laptev and Sannikov straits corresponds to the integration period of 9 days (Fig. 7a). On the other hand, no dependence was observed between salinity in the strait and the meridional Ekman transport $E_{N}^{y}$ averaged over Area 3 (Fig. 7b).

The obtained result shows that freshwater transport from the Laptev Sea to the East-Siberian Sea through the Laptev and Sannikov straits is directly determined by zonal Ekman transport of the core of the Lena plume in the south-eastern part of the Laptev Sea (Area 3) and not by the alongshore upwelling/downwelling winds, which is the case of freshwater transport within the narrow coastal buoyancy current through the Vilkitsky Strait. In particular, strong southerly wind induces intense eastward Ekman transport of the Lena plume and decrease salinity in the Laptev and Sannikov straits, while strong northerly wind, on the opposite, causes westward flow of the Lena plume and increase salinity in the straits. Strong northerly winds in the study area in the beginning of October 2016 induced intense westward Ekman transport ($E_{9}^{x}$ = − 5.8 m^2^/s day) that resulted in relatively large salinity (22) in the Laptev Strait registered on 8 October 2016 (Fig. 7a). This point is a distinct outlier in the analyzed set. However, it is not a measurement error, because its large salinity value is an average of more than 1,000 individual salinity measurements in the surface layer. We calculated two linear trend lines, namely, for the whole set of points and for the set without the outlier, the resulting lines are very similar (Fig. 7a). However, the Pearson correlation coefficient for the whole set (0.7) is greater that for the set without the outlier (0.5).

In situ measurements performed in September 2017 also revealed response of surface salinity in the Sannikov Strait on synoptic variability of wind forcing illustrated by red arrow in Fig. 7a. Salinity in the strait increased from 18 on 4 September 2017 to 21 on 13 September 2017 (Fig. 5a, b). Moderate easterly wind observed on 28 August–8 September switched to strong northerly wind on 9–13 September that induced intense westward Ekman transport in the study area. As a result, the zonal Ekman transport integral $E_{9}^{x}$ changed from − 0.1 m^2^/s day on 4 September 2017 to − 1.8 m^2^/s day on 13 September 2017.

## Discussion

In this study we focus on inter-basin freshwater transport between the Kara, Laptev, and East-Siberian seas associated with spreading of surface-advected river plumes, i.e. shallow (10–20 m deep) freshened (< 25) water masses with large salinity gradients with ambient sea. We show that this eastward transport is wind-driven and is characterized by quick response to synoptic variability of wind forcing. However, different configurations of freshwater transport pathways in the Kara and Laptev seas defined by distances between the main freshwater sources and the inter-basin straits, as well as topographic barriers result in significant differences in wind conditions that induce this transport.

The main branches of the Lena Delta inflow to the Laptev Sea on a distance of 350–400 km from the Laptev and Sannikov straits. The Lena plume occupies the area in the south-eastern part of the Laptev Sea adjacent to the straits and propagates eastward through them as a large-scale surface water mass. Due to strong vertical stratification between the Lena plume and the subjacent saline sea, vertical momentum flux across the bottom boundary of the Lena plume is presumed to be negligible. As a result, the Ekman transport determines the wind-driven spreading of the Lena plume on a distance comparable to spatial extents of the plume and far from the coastline^40,47^. Therefore, salinity in the Laptev and Sannikov straits that is indicative of intensity of freshwater transport from the Laptev Sea to the East-Siberian Sea is governed by eastward Ekman transport of the Lena plume and has very quick response (~ 5 to 10 days to variability of wind forcing conditions. According to the Ekman theory, strong northerly winds can slacken this freshwater transport; however, we are not aware of any in situ measurements (including those published in previous studies at this region that registered surface salinity > 22, i.e., absence of the Lena plume, in the Laptev and Sannikov straits during ice-free periods. The quantitative assessment of response of freshwater transport through these straits on wind forcing requires in situ velocity measurements and is within the scope of future work.

The Ob and Yenisei rivers inflow to the Kara Sea on a distance of 850–1,000 km from the Vilkitsky Strait. The Ob-Yenisei plume occupies the area in the central part of the Kara Sea and the distance between the core part of the plume and the Vilkitsky Strait exceeds 400–500 km which is registered by in situ measurements and satellite observations. The northeastward Ekman transport induces spreading of the Ob-Yenisei plume towards the Vilkitsky Strait, albeit this large-scale water mass does not reach the strait under even strong and long-term southeasterly winds. However, the Ob-Yenisei plume forms narrow buoyancy current that propagates along the Taymyr Peninsula towards the Vilkitsky Strait. In absence of wind forcing this current is geostrophic, flow in this current is enhanced by downwelling-favorable winds and hindered by upwelling-favorable winds, while the Ekman theory does not work for this current due to proximity of a coastline. Due to large distance between the core of the Ob-Yenisei plume and the Vilkitsky Strait, as well as multiple topographic barriers (capes and islands) along the Taymyr Peninsula, the freshened alongshore current reaches the Vilkitsky Strait only under strong and long-term (~ 25 to 30 days) southwesterly winds. This results in very high synoptic variability of surface salinity in the Vilkitsky Strait between the spreading (< 25) and non-spreading (> 28) periods of the Ob-Yenisei plume.

**Figure 8:**
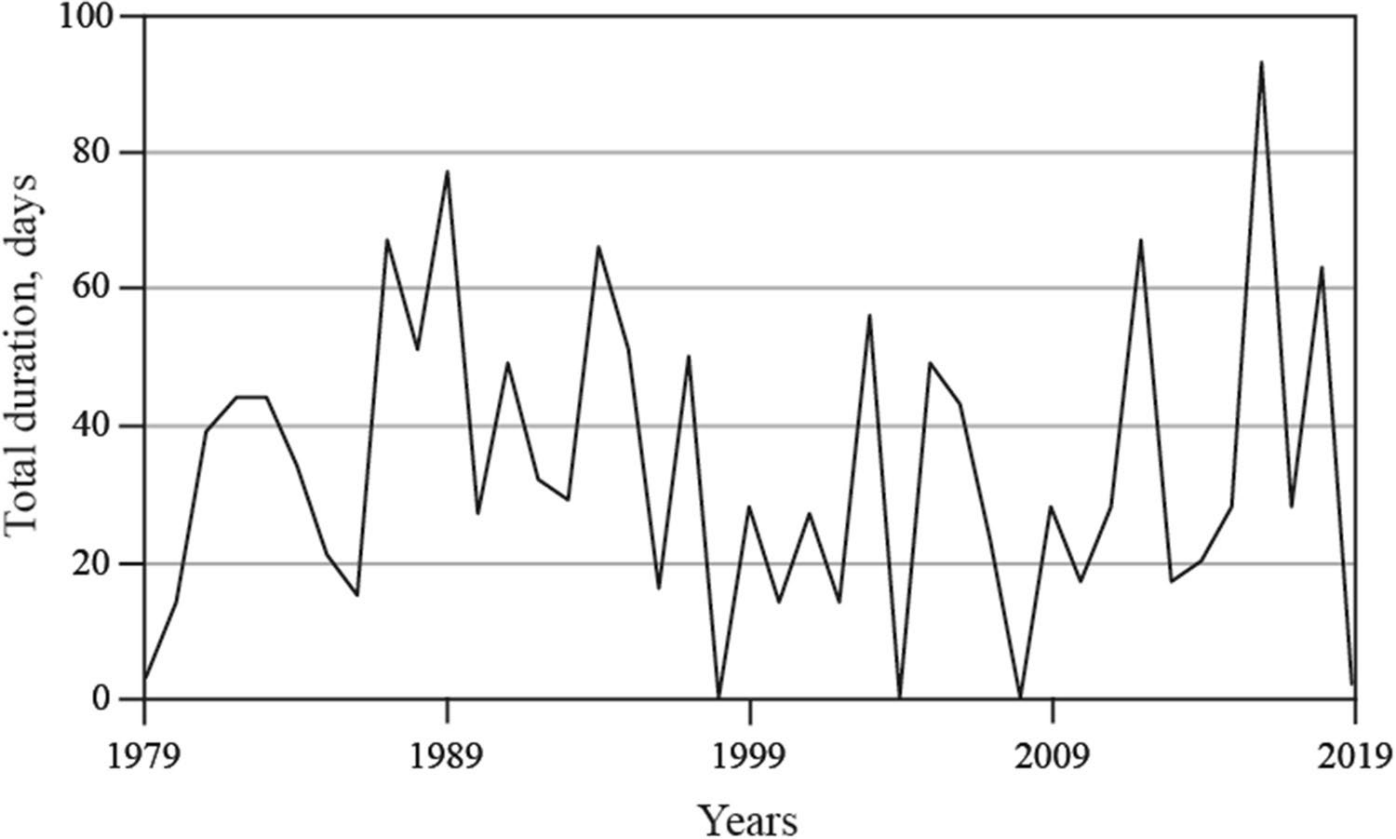
Total annual duration of spreading periods of the Ob-Yenisei plume to the Laptev Sea through the Vilkitsky Strait during ice-free periods in 1979–2019.

The Ob-Yenisei and Lena plumes are the main sources of low salinity water in the surface layer in the Kara and Laptev seas during ice-free periods. As during this season the impact of the sea ice melting on salinity in coastal areas is negligible^48^, variability of surface salinity in the Vilkitsky, Sannikov, and Laptev straits is indicative of eastward spreading of these river plumes. The analysis of wind forcing, salinity data, and satellite imagery shows that generally there are two salinity regimes in the Vilkitsky Strait (indicated by boxes in Fig. 4a), namely, low salinity (< 25) during spreading of the Ob-Yenisei plume to the Laptev Sea induced by strong and long-term southwesterly winds ($W_{26}^{x}$ > 10 m/s day) and high salinity during non-spreading periods. As a result, spreading periods of the Ob-Yenisei plume to the Laptev Sea during ice-free periods can be reconstructed using wind data. Using ERA5 wind reanalysis we calculated $W_{26}^{x}$ for every day for the ice-free seasons (15 July–15 October) in 1979–2019 and assessed periods of wind forcing favorable for eastward inter-basin spreading of the Ob-Yenisei plume. The total annual duration of spreading periods varied from 0 days in 1998 and 2008, i.e., negligible transport, to 67 days in 1987 and 2012, 77 days in 1989, and even 93 days in 2016, i.e., almost constant transport during the ice-free season (Fig. 8). The average annual duration of spreading periods in 1979–2019 was 34 days. This result demonstrates that frequency and duration of these spreading events have significant inter-annual variability, which can strongly influence stratification, ice formation, biological productivity, and many other related processes at the shelf areas in the Laptev Sea and the eastern Arctic Ocean.

The largest salinity (22), albeit small enough to be related to the Lena plume, was registered in Laptev and Sannikov straits on 8 October 2016 and was preceded by the strong northerly winds on 1–5 October that induced the strongest westward Ekman transport integral ($E_{9}^{x}$ = −5.8 m^2^/s day) among the analyzed data. More intense Ekman transport, i.e., $E_{9}^{x}$ < −6 m^2^/s day, was observed in 1979–2019 only in October 2016 several days before and after these in situ measurements. Therefore, we presume that the Lena plume was constantly present in the Laptev and Sannikov straits during ice-free seasons in 1979–2019.

The Ob, Yenisei, and Lena runoffs account for approximately a half of the total surface freshwater flux to the Arctic Ocean^1–3^. Spreading and transformation of the Ob-Yenisei and Lena plumes addressed in this study is important for understanding large-scale freshwater transport along the Eurasian Arctic coast that strongly affects stratification and ice formation, as well as many other physical, biological, and geochemical processes in the Arctic Ocean. The existing estimations of inter-annual variability of freshwater transport based on large-scale atmospheric forcing^13,25,28^ can be improved by assessment of synoptic wind variability during individual years.

The western shore of the Taymyr Peninsula and the Severnaya Zemlya archipelago effectively hinders spreading of the Ob-Yenisei plume to the Laptev Sea, which occurs only under specific wind forcing conditions. Moreover, there is no evidence that the Ob-Yenisei plume is spreading northward towards the central part of the Arctic Ocean during the ice-free season according to previous publications^15,16,33^, as well as available in situ data and satellite imagery. Therefore, large freshwater discharge from the Ob and Yenisei rivers is mainly accumulated within the Kara Sea during ice-free season of certain years. The Lena plume, on the opposite, is commonly spreading to the western part of the East-Siberian Sea during ice-free season. The Lena plume also forms a narrow freshened alongshore current in the eastern part of the East-Siberian Sea. This current is enhanced by freshwater discharges from the large Indigirka and Kolyma rivers and propagates further eastward to the Chukchi Sea in absence of coastal topographic barriers where it is referred as the Siberian Coastal Current^49–51^.

All in situ measurements used in this study were performed from the end of August to the middle of October. During these periods discharges of the Ob, Yenisei, and Lena rivers are relatively homogenous and shelf areas of the Kara and Laptev seas are free of ice^33^. Therefore, we do not assess influence of large seasonal variability of river discharge on inter-basin freshwater transport. Nevertheless, this transport can be more intense during freshet periods that occur in June–July and provide half of total annual discharge of the Ob, Yenisei, and Lena rivers. Also we do not consider inter-basin freshwater transport that can occur in late autumn, winter, and spring when the Kara, Laptev, and East-Siberian seas are covered by ice and influence of wind forcing on the dynamics of the Ob-Yenisei and Lena plumes is negligible. The qualitative results of this study provide the necessary background for organization of comprehensive year-round monitoring of freshwater transport between these seas using extended network of salinity and velocity measurements, which is essential for reconstructing seasonal, annual and inter-annual patterns and variability of large-scale freshwater transport in the Arctic Ocean.

## Data and methods

Hydrographic in situ data used in this study were collected during 15 oceanographic surveys in the Kara, Laptev, and East-Siberian seas of the research vessels “Dunay”, “Nikolay Kolomeytsev”, “Ivan Kireev”, “Auga”, “Kapitan Dranitsyn”, “Victor Buynitskiy”, “Yakov Smirnitskiy”, “Akademik Lavrentyev”, “Akademik Mstislav Keldysh” in 1999–2019 (Table 1). The field surveys included continuous measurements of salinity in the surface sea layer (2–3 m depth) performed at 100 m spatial resolution along the ship track using a ship board pump-through system equipped by a thermosalinograph instrument (*Sea-Bird Electronics SBE 21 SeaCAT*). These measurements were performed in the Kara, Laptev, and East-Siberian seas during nine field surveys in August–September 2008, September–October 2011, September 2012, September 2015, September–October 2016, September 2017, August–September 2018, September–October 2018, September–October 2019. Also we used vertical thermohaline profiles performed using a CTD instrument (*Sea-Bird Electronics SBE 911plus*) at 0.2 m vertical resolution. These measurements were performed in the Vilkitsky, Sannikov, and Laptev straits during seven field surveys in September 1999, August–September 2000, September 2003, September 2004, September 2005, September 2006, September 2018. Finally, we used thermohaline measurements in the surface layer (0–3 m depth) performed by autonomous salinity loggers (*Star-Oddi DST CTD*) mounted at the mooring station in the Vilkitsky Strait. These measurements were performed on 24 September–11 October 2019. Satellite maps of sea surface distributions of corrected reflectance and brightness temperature at the study areas were retrieved from MODIS satellite data by ESA BEAM software (version 5.0). The atmospheric influence on the Ob-Yenisei and Lena plumes was examined using 10-m winds and sea-level pressure fields from 1 h ERA5 atmospheric reanalysis with a 0.25° resolution.

**Table 1:** Dates, research vessels, areas, and type of in situ measurements of oceanographic surveys.

Dates	Research vessel	Area	Type of in situ measurements
12.09.1999	Dunay	Laptev Strait	Hydrographic stations
5.09.2000	Nikolay Kolomeytsev	Laptev Strait	Hydrographic stations
20.09.2003	Ivan Kireev	Laptev Strait	Hydrographic stations
01.09.2004	Ivan Kireev	Sannikov Strait	Hydrographic stations
7.09.2005	Auga	Vilkitsky Strait	Hydrographic stations
15.09.2005	Auga	Laptev Strait	Hydrographic stations
20.09.2005	Auga	Laptev Strait	Hydrographic stations
29.09.2006	Kapitan Dranitsyn	Vilkitsky Strait	Hydrographic stations
30.08.2008	Yakov Smirnitskiy	Laptev Strait	Continuous measurements and hydrographic stations
16.09.2008	Yakov Smirnitskiy	Sannikov Strait	Continuous measurements
19.09.2008	Yakov Smirnitskiy	Vilkitsky Strait	Continuous measurements
18.09.2011	Akademik Lavrentyev	Sannikov Strait	Continuous measurements and hydrographic stations
30.09.2011	Akademik Lavrentyev	Laptev Strait	Continuous measurements and hydrographic stations
8.09.2012	Victor Buynitskiy	Vilkitsky Strait	Continuous measurements
18.09.2012	Victor Buynitskiy	Laptev Strait	Hydrographic stations
25.09.2012	Victor Buynitskiy	Vilkitsky Strait	Continuous measurements
5.09.2015	Akademik Mstislav Keldysh	Vilkitsky Strait	Continuous measurements
8.10.2016	Akademik Lavrentyev	Laptev Strait	Continuous measurements
4.09.2017	Akademik Mstislav Keldysh	Sannikov Strait	Continuous measurements
13.09.2017	Akademik Mstislav Keldysh	Sannikov Strait	Continuous measurements
21.09.2017	Akademik Mstislav Keldysh	Vilkitsky Strait	Continuous measurements
23.08.2018	Akademik Mstislav Keldysh	Vilkitsky Strait	Continuous measurements
4–5.09.2018	Akademik Mstislav Keldysh	Vilkitsky Strait	Continuous measurements and hydrographic stations
26.09.2018	Akademik Mstislav Keldysh	Vilkitsky Strait	Continuous measurements
19.10.2018	Akademik Mstislav Keldysh	Vilkitsky Strait	Continuous measurements
24.09.2019	Akademik Mstislav Keldysh	Vilkitsky Strait	Continuous measurements and hydrographic stations
5.10.2019	Akademik Mstislav Keldysh	Laptev Strait	Continuous measurements and hydrographic stations
14.10.2019	Akademik Mstislav Keldysh	Vilkitsky Strait	Continuous measurements and hydrographic stations
24.09–14.10.2019	–	Vilkitsky Strait	Mooring station

## Supplementary information

Supplementary Legends.

Supplementary Information 1.

Supplementary Information 2.

Supplementary Information 3.

## Data Availability

The ERA5 reanalysis data were downloaded from the European Centre for Medium-Range Weather Forecasts (ECMWF) website https://www.ecmwf.int/en/forecasts/datasets/archive-datasets/reanalysis-datasets/era5. The MODIS Terra and Aqua satellite data were downloaded from the NASA repository of the satellite data https://ladsweb.modaps.eosdis.nasa.gov/. Satellite data was processed using the ESA BEAM software (version 5.0) available at https://www.brockmann-consult.de/cms/web/beam/releases. The in situ data are available in supplementary information.
