# Supplementary material for: Freshwater transport between the Kara, Laptev, and East-Siberian seas
Source: Sci Rep. 2020 Aug 3;10:13041. doi: 10.1038/s41598-020-70096-w (PMC7400578; doi:10.1038/s41598-020-70096-w)
Supplement: Supplementary file 1 — Supplementary Legends. [file 41598_2020_70096_MOESM1_ESM.docx]

**Supplementary information - mooring station**

Dates, time, coordinates, and surface salinity at the mooring station in the Vilkitsky Strait.

**Supplementary information - surface measurements**

Dates, time, coordinates, and surface salinity of continuous measurements along ship tracks in the Vilkitsky, Laptev, and Sannikov straits.

**Supplementary information - vertical profiles**

Dates, time, coordinates, depth, and salinity of measurements at hydrographic stations in the Vilkitsky and Laptev straits.
